# Case report: Physical findings, physical therapy practice, and characteristics of disability of activities of daily living caused by obturator nerve palsy after neurotmesis

**DOI:** 10.3389/fneur.2023.1062018

**Published:** 2023-01-24

**Authors:** Daichi Shima, Tokio Kinoshita, Yasunori Umemoto, Yoshinori Yasuoka, Takamasa Hashizaki, Makoto Asaeda, Yukihide Nishimura, Tamaki Yahata, Takashi Shimoe, Fumihiro Tajima

**Affiliations:** ^1^Department of Rehabilitation Medicine, Wakayama Medical University, Wakayama, Japan; ^2^Division of Rehabilitation, Wakayama Medical University Hospital, Wakayama, Japan; ^3^Faculty of Wakayama Health Care Sciences, Takarazuka University of Medical and Health Care, Wakayama, Japan; ^4^Department of Rehabilitation Medicine, Iwate Medical University, Shiwa-gun, Japan; ^5^Department of Obstetrics and Gynecology, Wakayama Medical University, Wakayama, Japan; ^6^Department of Orthopaedic Surgery, Wakayama Medical University, Wakayama, Japan

**Keywords:** obturator nerve, peripheral nerve injury, activities of daily living, rehabilitation, case report

## Abstract

The obturator nerve originates from the lumbar plexus and innervates sensation in the thigh and movement of the adductor muscle group of the hip. Reports on physical therapy for patients with obturator nerve injuries have been limited due to insufficient injuries, and there have been no reports on rehabilitation after neurotmesis. Furthermore, there are no reports on the status of activities of daily living (ADL) and details of physical therapy in patients with paralysis of the adductor muscle group. In this study, we reported on a patient with adductor paralysis due to obturator neurotmesis, including the clinical symptoms, characteristics of ADL impairment, and effective movement instruction. The patient is a woman in her 40's who underwent laparoscopic total hysterectomy, bilateral adnexectomy, and pelvic lymph node dissection for uterine cancer (grade-2 endometrial carcinoma). During pelvic lymph node dissection, she developed an obturator nerve injury. She underwent nerve grafting during the same surgery by the microsurgeon. Donor nerve was the ipsilateral sural nerve with a 3-cm graft length. Due to obturator nerve palsy, postoperative manual muscle test results were as follows: adductor magnus muscle, 1; pectineus muscle, 1; adductor longs muscle, 0; adductor brevis muscle, 0; and gracilis muscle, 0. On postoperative day 6, the patient could independently perform ADL; however, she was at risk of falling toward the affected side when putting on and taking off her shoes while standing on the affected leg. The patient was discharged on postoperative day 8. Through this case, we clarified the ADL impairment of a patient with adductor muscle palsy following obturator neurotmesis, and motion instruction was effective as physical therapy for this disability. This case suggests that movement instruction is important for acute rehabilitation therapy for patients with hip adductor muscle group with obturator neurotmesis.

## 1. Introduction

The obturator nerve arises from the anterior part of the ventral branches of the second, third, and fourth lumbar nerves of the lumbar plexus, and provides sensory innervation of the hip joint and mid-thigh, and motor innervation of the adductor muscles (1). The obturator nerve is often damaged by surgery, hemorrhage, tumor compression, and sports-related trauma (2–7). Symptoms include pain in the medial groin area, loss of sensation in the medial thigh, and weakness of the ipsilateral adductor muscle (2).

The severity of nerve damage, regardless of whether it is an obturator nerve, is divided into five grades by the Sunderland classification (8). While treatment and prognosis after nerve injury depend on injury severity, rehabilitation is often provided for all. Reports on rehabilitation in obturator nerve injury are limited. Yikilmaz et al. reported hospital discharge within 5–9 days of occurrence in three patients with clipping, one incomplete cut, and two complete transection obturator nerve injuries. However, details regarding activities of daily living (ADL) status or rehabilitation were not described (9). The most severe type of injury (grade V: the epineurium is torn, and nerve continuity is severed) is not expected to recover spontaneously, and thus, requires nerve repair or nerve grafting, and has the most severe functional disability (8, 10).

In this report, we describe a patient with a grade-V obturator nerve injury, with the characteristics of ADL impairment in the early disease stages, and details of effective physical therapy.

## 2. Case report

The patient was a woman in her 40's. Her body mass index was 23.9 kg/m^2^. Her current medical history included an infertility-associated hysteroscopy at a community hospital in 2008. During the procedure, a polyp was discovered and resected, and she was diagnosed with endometrial carcinoma G1 *via* pathological examination. She was referred to our university hospital for endometrial curettage, and no findings suggestive of hyperplasia or cancer were determined. In December 2021, she experienced excessive and prolonged menstruation. She presented to a regional hospital, where a transvaginal echocardiogram revealed endometrial polyp-like lesions, and she was referred back to our hospital for further examination and treatment. In January 2022, her tumor marker carbohydrate antigen 125 was normal at 7.4 U/mL. Imaging studies showed a borderline lesion with minimal or no myometrium invasion. She was diagnosed with stage IA, a low-risk recurrence group with absent distant metastasis, and was admitted to our hospital in March 2022 for surgical management of uterine cancer. The following day, she underwent laparoscopic total hysterectomy, bilateral adnexectomy, and pelvic lymph node dissection. She sustained a right obturator nerve transection (Sunderland classification: grade-V injury) during lymph node dissection and was referred to the orthopedic microsurgeon who performed a right sural nerve grafting (the defect of the obturator nerve was 3 cm, and the sural nerve grafting was performed in a cable graft manner). Three days after surgery, the patient was referred to our department for rehabilitation. Physical therapy was initiated on the same day.

**Figure 1:**
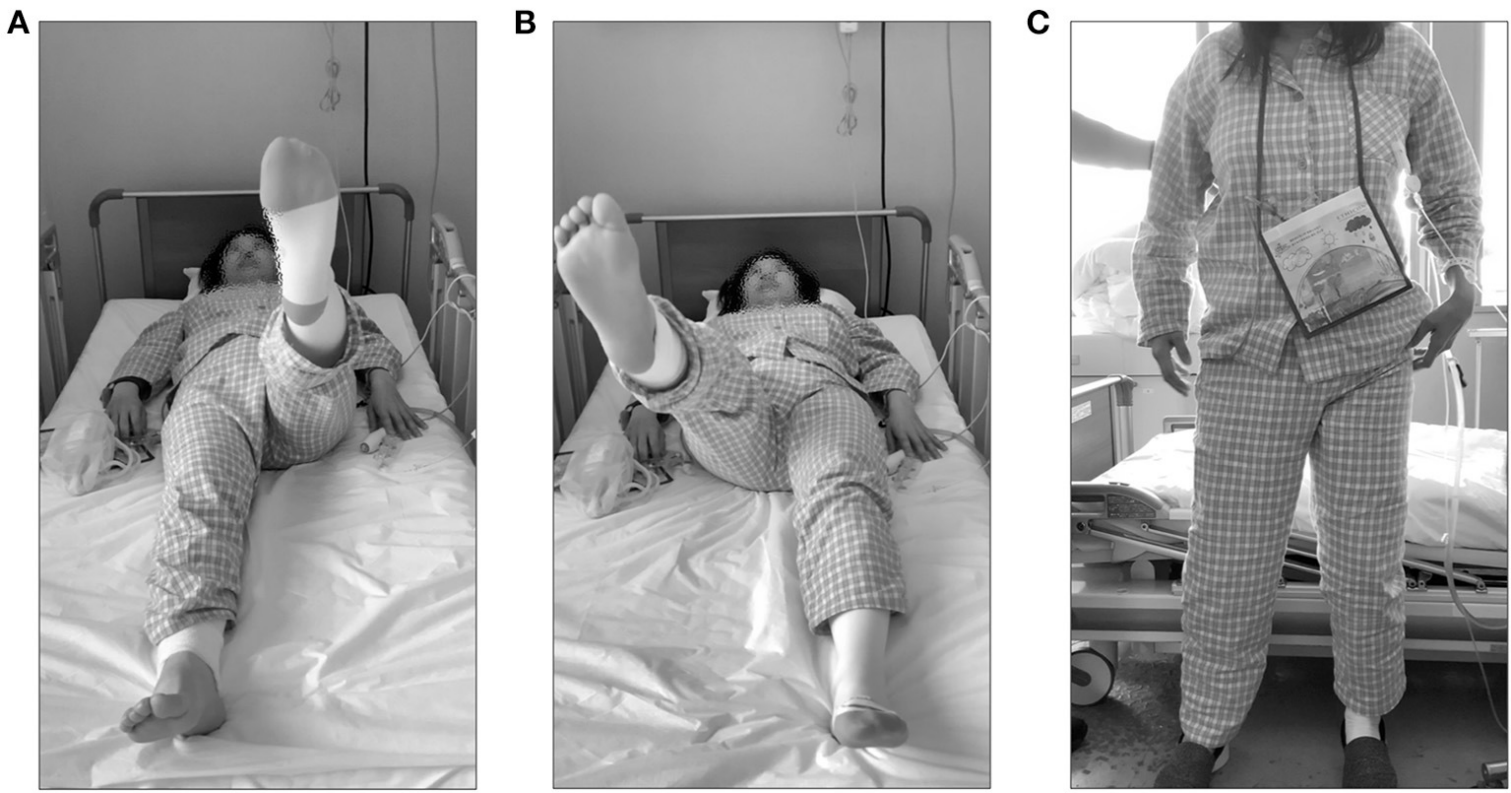
Visual examination due to obturator nerve injury. Visual examination performed on day 1 of postoperative intervention. **(A)** Straight leg raising with the healthy lower limb. **(B)** Straight leg raising with the affected lower extremity. **(C)** Standing posture with hip abduction and external rotation.

**Figure 2:**
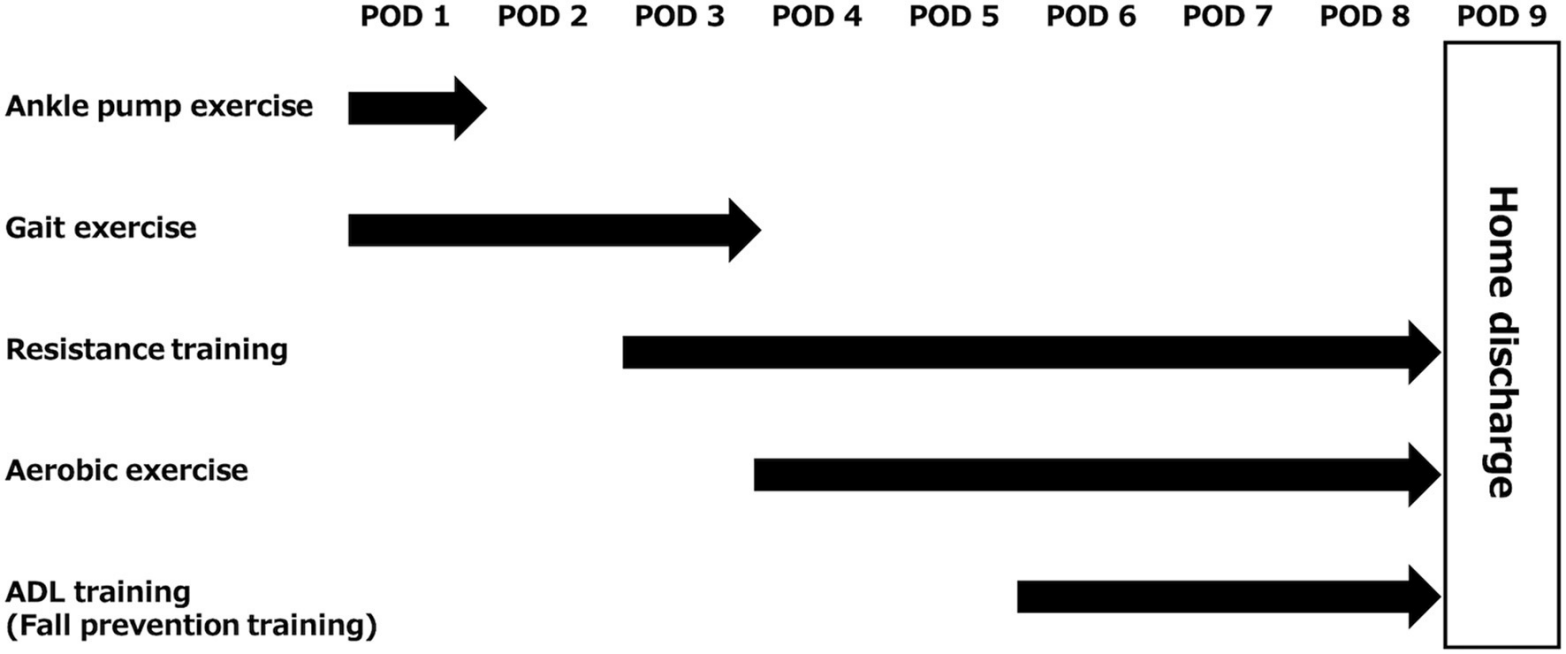
Description of rehabilitation for this case. The flow from rehabilitation initiation to home discharge was presented. ADL, activities of daily living; POD, postoperative day.

Vital signs at the initiation of rehabilitation were as follows: blood pressure, 101/69 mmHg; heart rate, 85 bpm; and SpO_2_, 97%. Her ocular conjunctivae were pallor-free, and no heart murmurs or pathological lung sounds were noted. Postoperative white blood cell count and C-reactive protein level were 10,380 μL and 5.85 mg/dL, respectively. The Glasgow Coma Scale was E4V5M6 (11, 12). Since the patient was bedridden on the first postoperative day, the evaluation was performed in bed. Patient-controlled analgesia was placed in the back, a wound drain in the abdomen, and an indwelling bladder catheter was inserted. Endoscopic and sural nerve harvesting wounds were visible in the lower abdomen and posterior right lower leg, respectively. The range of motion was confirmed to be 90° hip flexion and 10° abduction, and no other significant limitations were observed. No further motion was performed to avoid stretching the sutured nerves. Manual muscle testing (13) showed that the adductor muscles on the side of the obturator nerve injury were 1 for the adductor magnus and pectineus muscles and 0 for the adductor longs, adductor brevis, and gracilis muscles (Table 1). Sensory perception was not decreased in the obturator nerve area; however, there was sensory insensitivity and abnormal sensation in the sural nerve area. In the hip flexion exercise and straight leg raising on the bed, as compared with the healthy side (Figure 1A), hip abduction and external rotation occurred with hip flexion on the affected side (Figure 1B). The patient was unable to perform hip adduction at 0° hip extension and was gradually repositioned into adduction by flexing the knee joint during hip external rotation, followed by hip internal rotation and knee extension as a compensatory action. The functional independence measure was 54 points due to bed rest (Table 1). The patient was instructed to perform ankle pump exercises to prevent deep vein thrombosis (Figure 2).

**Table 1:** Postoperative changes in manual muscle testing (MMT) scores.

	**POD1**	**POD5**	**POD9**
FIM	54	125	126
MMT			
Pectineus	1	1	1
Adductor longs	0	0	0
Adductor brevis	0	0	0
Adductor magnus	1	1	1
Gracilis	0	0	0

FIM, functional independence measure; MMT, manual muscle testing; POD, postoperative day.

On postoperative day 2, after the resting level was changed to unrestricted, and hip range of motion exercises were unrestricted. Therefore, the patient was weaned off bed rest. The sitting position was stable and possible. The patient was able to stand independently; however, the hip joint on the affected side was mildly abducted and externally rotated (Figure 1C). The patient was able to walk unsupervised and experienced mild discomfort in the hip joint. To prevent secondary complications, such as deep vein thrombosis, bowel obstruction, and pneumonia, gait training was the focus of the early postoperative period. Then, low load aerobic and resistance training were gradually added, depending on the patient's condition (Figure 2).

On postoperative day 5, all routes were removed and the patient became independent. Muscle strength remained unchanged (Table 1). However, the hip abduction and external rotation that occurred during hip flexion on the bed were no longer present. The stairs also required handrails, and the functional independence measure was 125 points (Table 1). Thereafter, exercise therapy consisted of aerobic exercise on an ergometer at a moderate load, with additional stair climbing training. Resistance training consisted of squats with a ball between the legs, as well as heel raises, leg presses, bridges, and forward lunges (Figure 2).

**Figure 3:**
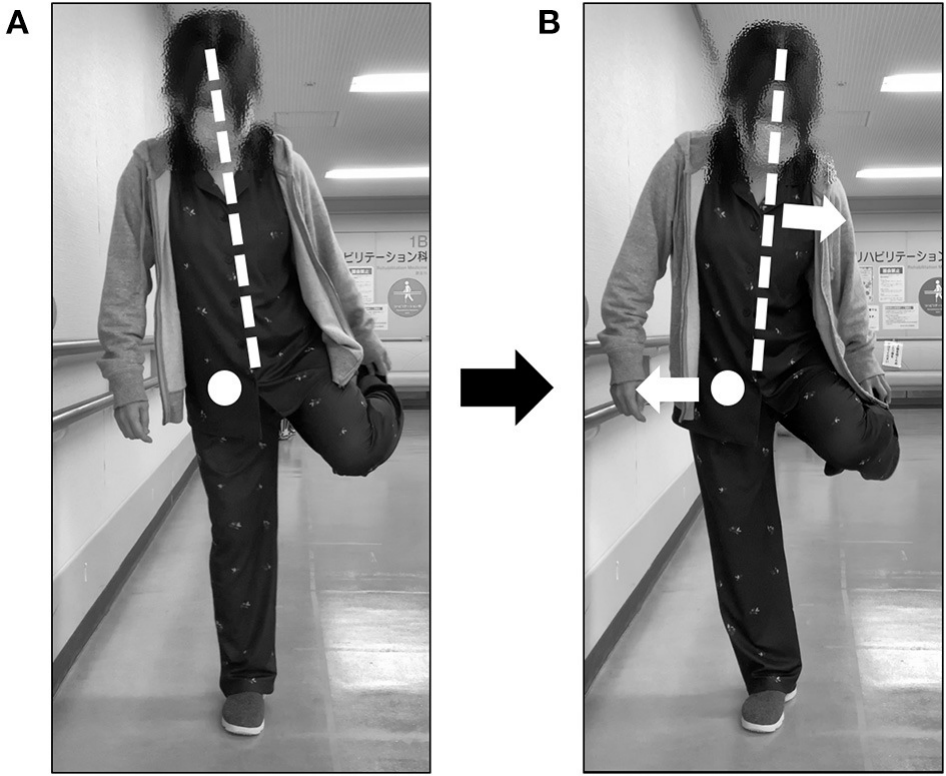
Points for which motion instruction was provided. **(A)** A posture with a risk of falling was indicated. **(B)** The posture of the patient after movement instruction was given. Trunk alignment was indicated by a dotted line and the hip axis was indicated by a circle. Arrows indicated the movement direction from the posture before the change **(A)**.

On postoperative day 6, the patient's movements were reviewed in preparation for home discharge and ADL training was then initiated (Figure 2). There was no risk of falling during basic activities. During one-leg standing, the unaffected side could hold the position for more than 15 s. However, on the affected (right) side, the trunk was flexed to the right within 2 s and she almost fell to that side. At that time, the manual muscle testing of the midline muscles was 5-/5. The patient's manual muscle testing was 5-/5 at the time of the injury. When the motion was reviewed, there was a similar risk of falling to the right side. In this case, she flexed her trunk to the right, abducted her left upper and lower limbs, and internally rotated her ankle joint (Figure 3A), to maintain her center of gravity inside the axis of motion of her hip joint; however, she still fell. To understand the degree to which the body's center of gravity did not exceed the axis of motion of the hip joint, the patient was additionally trained to shift her weight to the right in a one-legged standing position. The duration of the one-leg standing position was prolonged, yet the risk of falling remained. The patient was instructed to move the pelvis to the right and flex the trunk slightly to the left (Figure 3B).

On postoperative day 8, the patient was able to stand on one leg, change clothes, and put on and remove shoes. Finally, when the patient was about to fall, stepping was reviewed. Stepping was possible in the front, back, left, and right.

On postoperative day 9, all sutures were removed from the lower abdomen and right lower leg wounds. Muscle strength was unchanged (Table 1). Her functional independence measure was 126 points, and she was discharged home (Table 1). Additionally, loxoprofen was administered for pain, but no medication was used to improve the patient's recovery. In this case, an obstetrician/gynecologist and an orthopedic surgeon were scheduled to see the patient every 2 months and every 6 months, respectively, at our clinic. Therefore, we had the rehabilitation department come in each time to palpate the patient and examine the situation in her home. At the first time, there was no improvement in paralysis, but we were able to confirm that the patient was independent in all aspects of home life, with no falls.

## 3. Discussion

In this study, we highlighted ADL impairment seen in patients with obturator nerve injury, including the risk of falling, in activities that require one-leg standing and showed an effective method of motion instruction for these patients. To our best knowledge, there are no reports on the rehabilitation treatment of patients with sural nerve transplantation for a grade-V obturator nerve injury. This case report is the first to describe in detail the implementation of rehabilitation therapy in a patient with adductor muscle group paralysis due to an obturator nerve injury.

The patient had obvious postoperative paralysis of the adductor muscle group due to an obturator nerve injury that occurred during obstetric and gynecological surgery. In addition, the patient required compensatory movement for adduction on the bed. As rehabilitation treatment progressed, the risk of falling toward the paralyzed side was higher in movements that required standing on the leg of the paralyzed side. However, there were no reports investigating the risk of falling in patients with obturator nerve injury. When shifting from a bipedal to a one-leg standing posture, it is necessary to shift the position of the body's center of gravity to the stance leg side and maintain that position in the basal plane of support (14). Pelvic shift or trunk lateral bending is required to shift the center of gravity to the stance side. In our case, the patient was responsive and unable to maintain the one-leg upright posture. Therefore, she was at risk of falling to the one-leg upright side. This might result from excessive hip abduction torque generated by the continued shift of the body's center of gravity to the stance leg side (lateral), which moved the hip joint outward from the axis of motion. In healthy subjects, the hip adductor muscles of the stance leg side contract to maintain the one-leg standing position, producing hip adduction torque that can antagonize the hip abduction torque caused by the outward shift of the center of gravity (15). However, our patient was unable to generate hip adduction torque due to adductor paralysis, leading to a risk of falling to the paralyzed side. Therefore, as rehabilitation treatment, we instructed the patient to move in a posture that reduces hip abduction torque by utilizing the position of the body's center of gravity (Figure 3B). Soon after movement instruction, the patient's time in the one-legged standing position was prolonged, and 2 days later, she was able to move in the one-legged standing position. She was discharged home with a reduced risk of falling after movement instruction as acute rehabilitation treatment was provided. However, in order for the aforementioned instructions to be properly implemented, the patient must be able to maintain the abductor muscle strength of the paralyzed side of the hip, have no range of motion limitations in the hip, and be able to properly understand the instructions. Elderly patients often show muscle weakness with aging and have limited range of motion due to osteoarthritis. Additionally, their cognitive function is often impaired, and the instructional content may not be appropriate as in this case. In such cases, instructing the patient to hold a cane on the paralyzed side to antagonize the hip abduction torque may be one option. Furthermore, having the patient hold the luggage or other weights with the healthy upper extremity can also reduce the occurrence of abduction torque on the affected hip because the body center of gravity is shifted more medially. Thus, therapists need to choose appropriate instructional content for the subject.

In this case, a sural nerve graft was used for a grade-V injury of the obturator nerve. A sural nerve graft is commonly used because of its low sequelae after harvesting (16). A previous report concluded that the sural nerve was a good grafting material, as it demonstrated functional recovery during a follow-up period of 2 years when 12 nerve grafts were performed using a sural nerve (17). Conversely, a 71-year-old man with an obturator nerve transection and thermal injury who underwent sural nerve grafting reported reduced adductor muscle weakness after 6 months. Additionally, persistent sensory disturbance and muscle atrophy were observed in the lower limbs (9). Recent reviews have identified microsurgical techniques, patient age, lesion level, associated disease, and mechanism of injury as determinants of outcomes after nerve reconstruction (16, 18), and older age and more complex injury mechanism were poor prognostic factors. Because the patient was young and had no associated diseases, and the mechanism of injury was a sharp amputation, her prognosis was considered good. As the nerve recovery rate is 1–4 mm per day (19) and the distance from the injury site to the neuromuscular junction in this case was approximately 30 cm, recovery was expected to take more than half a year. Therefore, strengthening the residual muscles is necessary as a rehabilitation treatment. In the acute phase, when there is little functional improvement after a grade-V injury, it is important to provide movement guidance to avoid the risk of falling. Further periodic examinations should be conducted to confirm that the patient is maintaining the instructional program and is not falling down. Then, as soon as contraction of the obturator nerve innervating muscles is confirmed, a new rehabilitation treatment program should be offered to improve function.

Patients with obturator nerve injury were at risk of falling when moving in a one-leg standing position with a paralyzed hip adductor muscle group, indicating the requirement for motion guidance to reduce hip abduction torque using the center of gravity during movement. This study shows that movement instruction is important for acute rehabilitation treatment of patients with obturator nerve injury, due to a grade-V injury.

## Data availability statement

The raw data supporting the conclusions of this article will be made available by the authors, without undue reservation.

## Ethics statement

Ethical review and approval was not required for the study on human participants in accordance with the local legislation and institutional requirements. The patients/participants provided their written informed consent to participate in this study. Written informed consent was obtained from the individual(s) for the publication of any potentially identifiable images or data included in this article.

## Author contributions

All authors listed have made a substantial, direct, and intellectual contribution to the work and approved it for publication.
